# *ESR1*, *PGR*, *ERBB2,* and *MKi6*7 mRNA expression in diagnostic core biopsies from breast cancer patients of the ABCSG Trial 34

**DOI:** 10.1016/j.breast.2025.104633

**Published:** 2025-10-30

**Authors:** Stephanie Kacerovsky-Strobl, Christine Deutschmann, Dominik Hlauschek, Zsuzsanna Bago-Horvath, Christian F. Singer, Rupert Bartsch, Richard Greil, Karl Sotlar, Gabriel Rinnerthaler, Edgar Petru, Sigurd F. Lax, Daniel Egle, Angelika Pichler, Koppány Bodó, Andreas L. Petzer, Farid Moinfar, Jodi Weidler, Michael Bates, Peter Dubsky, Michael Gnant, Martin Filipits

**Affiliations:** a Department of Surgery, Klinikum Donaustadt, Vienna, Austria; b Austrian Breast and Colorectal Cancer Study Group (ABCSG), Vienna, Austria; c Department of Obstetricss and Gynecology, Division of Oncology, Breast Health Center and Comprehensive Cancer Center, Medical University of Vienna, Vienna, Austria; d Department of Pathology, Division of Oncology, Breast Health Center and Comprehensive Cancer Center, Medical University of Vienna, Vienna, Austria; e Department of Medicine I, Division of Oncology, Breast Health Center and Comprehensive Cancer Center, Medical University of Vienna, Vienna, Austria; f 3rd Medical Department, Salzburg Cancer Research Institute, Cancer Cluster Salzburg, Paracelsus Medical University Salzburg, Salzburg, Austria; g Institute of Pathology, Paracelsus Medical University Salzburg, Salzburg, Austria; h Departments of Division of Oncology, Department of Internal Medicine, Medical University Graz, Graz, Austria; i Department of Gynecology and Obstetrics, Medical University Graz, Graz, Austria; j Department of Pathology, Hospital Graz II, Graz, Austria; k Johannes Kepler University Linz, Linz, Austria; l Department of Gynecology and Obstetrics, Medical University Innsbruck, Innsbruck, Austria; m Departments of Hemato-Oncology, LKH Hochsteiermark-Leoben, Leoben, Austria; n Departments of Pathology, LKH Hochsteiermark-Leoben, Leoben, Austria; o Departments of Internal Medicine I, Hematology with Stem Cell Transplantation, Hemostaseology and Medical Oncology, Ordensklinikum Linz Barmherzige Schwestern, Elisabethinen, Linz, Austria; p Departments of Pathology, Ordensklinikum Linz Barmherzige Schwestern, Elisabethinen, Linz, Austria; q Cepheid, Sunnyvale, CA, USA; r St. Anna Breast Center, Hirslanden Klinik St. Anna, Lucerne, Switzerland; s University of Lucerne, Faculty of Health Sciences and Medicine, Lucerne, Switzerland; t Comprehensive Cancer Center, Medical University of Vienna, Vienna, Austria; u Center for Cancer Research, Breast Health Center and Comprehensive Cancer Center, Medical University of Vienna, Vienna, Austria

**Keywords:** Breast cancer, mRNA, Immunohistochemistry, GeneXpert, STRAT4

## Abstract

**Aim:**

To investigate the performance of the Xpert® Breast Cancer STRAT4 (CE-IVD∗) Assay (STRAT4) in the neoadjuvant, randomized Austrian Breast and Colorectal Cancer Study Group (ABCSG) Trial 34.

**Patients and methods:**

The primary objective of this study was to assess the concordance between STRAT4 mRNA measurements of *ESR1*, *PGR*, *ERBB2*, and *MKi67* obtained from diagnostic core biopsies of the ABCSG Trial 34 with central reference laboratory immunohistochemistry (IHC) (and in-situ hybridization for HER2 IHC 2+ samples). For each marker, overall percent agreement (concordance), positive percent agreement (sensitivity), and negative percent agreement (specificity) between STRAT4 and IHC were determined. The secondary objective was to evaluate the concordance of STRAT 4 and IHC in post-treatment surgical samples and the association of both assays with residual cancer burden (RCB), time to distant recurrence (DR), and overall survival (OS). Logistic regression and Cox regression models were used.

**Results:**

A total of 354 formalin-fixed paraffin-embedded diagnostic core biopsies were examined, representing 88.5 % of the available samples. Concordance between STRAT 4 and IHC was 93.7 % for ER, 80.5 % for PR, and 94.1 % for Ki67. All three biomarkers tested by either STRAT4 or central IHC showed similar correlation to RCB, time to DR, and OS.

**Conclusions:**

In diagnostic core biopsies, there was a good agreement between STRAT4 mRNA measurements and centrally assessed IHC measurements of ER, and moderate agreement for PR and Ki67.

## Highlights

• Immunohistochemistry (IHC) is the gold standard for evaluating ER, PR, HER2, and Ki67.
• The Xpert® Breast Cancer STRAT4 Assay represents an alternative diagnostic method.
• Assay performance was evaluated on diagnostic core biopsies and on surgical samples.
• We demonstrated good or moderate agreement between STRAT4 mRNA measurements and central IHC.

## 1 Introduction

Biomarkers including estrogen receptor (ER), progesterone receptor (PR), human epidermal growth factor receptor 2 (HER2), and the proliferation marker Ki67 have prognostic and predictive value [1,2] in early breast cancer. In routine clinical practice, the expression of these markers is assessed by immunohistochemistry (IHC) [[3], [4], [5]]. In HER2 IHC 2+ (equivocal) cases, the status of *ERBB2* copy number is additionally determined by in situ hybridization (ISH) [4]. Intra- and inter-observer variability of IHC of the four biomarkers, particularly Ki67, has been critically discussed, emphasizing the need for new standardized and more quantitative analytical methods [[4], [5], [6]].

Tests based on mRNA expression level are a potential alternative to IHC. The Xpert® Breast Cancer STRAT4 Assay (STRAT4) is a CE-IVD^2^ labeled *in vitro* diagnostic medical device. It is a cartridge-based test performed on the GeneXpert® Instrument platform that automates nucleic acid extraction and purification and real-time quantitative reverse transcription polymerase chain reaction (RT-qPCR) detection of target genes (*ESR1* [NCBI Entrez Gene ID: 2099], *PGR* [NCBI Entrez Gene ID: 5241], *ERBB2* [NCBI Entrez Gene ID: 2064]*,* and *MKi67* [NCBI Entrez Gene ID: 4288]) and a control gene (*CYFIP1*) mRNA in formalin-fixed, paraffin-embedded (FFPE) tissue. The system uses single-use disposable cartridges that hold the on-board PCR reagents and host the nucleic acid extraction, purification, and PCR process [7]. We have shown previously that this technology offers similar analytical performance to IHC, but at a lower time and cost [8].

In this study, we investigated whether the STRAT4 test is comparable to centrally assessed IHC in its analytical performance and prognostic value using diagnostic core biopsies and post-treatment surgical resection specimens of the neoadjuvant, randomized phase II Austrian Breast & Colorectal Cancer Study Group Trial 34 (ABCSG Trial 34) [9].

## 2 Patients and Methods

### 2.1 ABCSG Trial 34

ABCSG Trial 34 was a multicenter prospective randomized open-label phase II trial investigating the efficacy and safety of tecemotide, a cancer vaccine targeting glycoprotein mucin-1 (MUC-1), when added to neoadjuvant standard-of-care treatment (neoadjuvant chemotherapy [NaCT] or neoadjuvant endocrine therapy [NET]) in early breast cancer patients [9]. A total of 400 patients were included in the trial, 311 patients in the NaCT cohort and 89 patients in the NET arm. Premenopausal women, women with triple-negative breast cancer, patients with low or absent ER expression, and patients with G3 tumors received four cycles of epirubicin/cyclophosphamide plus four cycles of docetaxel with or without tecemotide.

Postmenopausal women with high or intermediate ER expression, and Ki67 < 14 %, and G1/2 tumors (luminal A-like tumors) received 6 months of letrozole with or without tecemotide. No significant difference in residual cancer burden (RCB) 0/I rates between patients with and without tecemotide in the overall study population nor in endocrine or chemotherapy-treated subgroups was observed [9,10]. Furthermore, no differences in the overall pathologic complete response (pCR) rates, MUC-1 expression, or tumor-infiltrating lymphocytes content were observed with the addition of tecemotide [9]. The study was conducted in accordance with the Declaration of Helsinki, approved by the responsible ethics committees, and all patients gave written informed consent.

### 2.2 Specimen collection and immunohistochemistry

FFPE tissue samples were collected at baseline (treatment-naïve) as well as at the time of surgery (treatment-exposed). One hematoxylin/eosin-stained (H&E) slide was prepared from each paraffin block and examined by an experienced breast pathologist to confirm the presence of invasive breast cancer. Adjacent unstained 4 μm FFPE tissue sections were prepared for ER (clone SP1; Roche-Ventana), PR (clone 1E2; Roche-Ventana), HER2 (clone 4B5; Roche-Ventana), and Ki67 (clone 30-9; Roche-Ventana) IHC on a BenchMark ULTRA® system (Roche-Ventana). Furthermore, in cases with HER2 IHC 2+ results additional tissue sections were used for ISH (INFORM HER2 dual probe; Roche-Ventana) on the BenchMark ULTRA® system. All IHC testing was centrally performed in an academic reference laboratory at the Center for Cancer Research, Medical University of Vienna and evaluated according to current ESMO and ASCO/CAP guidelines by an experienced breast pathologist blinded to the clinical outcome [[4], [5], [6]].

All invasive tumor cells on each slide were reviewed by visual assessment and interpretation of the results was limited to the invasive part of the tumor. For ER, PR, and Ki67, only nuclear staining was interpreted as positive, regardless of the staining intensity. The results were reported as the percentage of ER-, PR-, and Ki67-stained nuclei. The positive cutoff was ≥1 % or ≥1 % stained nuclei for ER and PR. A Ki67 value of ≤5 % was considered low, ≥30 % was considered high [6]. Patients with a Ki67 > 5 % and <30 % were excluded for the primary objective. HER2 IHC 3+ was considered positive, in HER2 IHC 2+ cases ISH analysis was performed and samples were considered positive according to the current ASCO/CAP guidelines [4].

### 2.3 Xpert® breast cancer STRAT4 assay

The STRAT4 assay allows the simultaneous detection of *ESR1*, *PGR*, *ERBB2*, and *MKi6*7 mRNA relative to the expression level of a control gene, *CYFIP1*. The STRAT4 test cartridge enables nucleic acid purification, amplification, and real-time detection and quantification by PCR of mRNA targets by using a fully automated and completely integrated system after lysate sample preparation.

For preparation of the lysate, one complete unstained FFPE section with tumor tissue cut at 10 μm was placed at the bottom of a 1.5 ml tube. If the FFPE section contained <30 % invasive tumor, macro-dissection was performed and tumor areas were scraped off the slide. In cases with extensive intraductal component, DCIS was removed prior to performing the assay.

After adding 1.2 ml of FFPE lysis buffer and 20 μl of proteinase K (Xpert® FFPE Lysis Kit, CE-IVD), the sample was mixed with a vortex mixer at maximum setting continuously for 10 s and incubated at 80 °C for 30 min. After being vortexed for 5 s and briefly spun-down for 3 s, the sample was transferred to a 5 ml sample container and 1.2 ml of 95 % ethanol was added. 520 μl of the lysate was transferred to the Xpert STRAT4 cartridge for each sample. The filled cartridge was placed in the on-demand real time-qPCR GeneXpert Instrument system (GX system, Cepheid, Sunnyvale CA, USA) and the assay was started. The instrument runtime for the test is approximately 70 min. For cases with invalid *CYFIP1* (cycle threshold (Ct) value > 35), a more concentrated tissue lysate was prepared according to the concentrated lysate sample preparation instructions per the manufacturer Xpert® FFPE lysis kit package insert instructions.

The cartridge test results were reported as ΔCt measurements (ΔCt = *CYFIP1* Ct – target gene Ct). The prespecified ΔCt thresholds for overexpression (positive results) relative to *CYFIP1* were ≥ −1.0 for *ESR1*, ≥ −3.5 for *PGR*, ≥ −1.0 for *ERBB2*, and ≥ −4.0 for *MKi67.* Any *CYFIP1* Ct value > 35 was considered insufficient to generate valid test results. The *CYFIP1* Ct cutoff for *ESR1* and *ERBB2* was set at ≤35. To minimize the rate of false-negative results for *PGR* and *MKi67,* a more stringent *CYFIP* cutoff of 31 was applied when the ΔCt values for *PGR* or *MKi67* were below the prespecified ΔCt positivity cutoffs (ΔCt < −3.5 for *PGR* or *<* −4.0 for *MKi67*). In these cases, where *CYFIP1* Ct > 31 and the ΔCt values for *PGR* or *MKi67* were below the cutoff, the result was reported as *PGR* or *MKi67* indeterminate rather than negative, indicating that the minimum assay input criteria were not met and the test should be repeated by adding are more concentrated lysate to the cartridge to achieve a *CYFIP1* Ct of at least 31.

### 2.4 Statistical analysis

All data analyses were performed according to a predefined statistical analysis plan and separately for the NaCT and the NET cohort. The primary objective of the study was concordance between STRAT4 mRNA measurements of *ESR1*, *PGR*, *ERBB2*, and *MKi67* with central reference laboratory IHC (and ISH for HER2 IHC 2+ cases) in diagnostic core biopsies. For the concordance analysis, 2 by 2 tables were created for each marker versus IHC/ISH as the reference and 95 % confidence intervals (CIs) were generated using the Wilson-Score method. The predefined ΔCt values (relative to control Ct values) of each marker were used to define the positive/negative call for each category of the breast cancer subtypes. Overall percent agreement (OPA) (concordance), positive percent agreement (PPA) (sensitivity), negative percent agreement (NPA) (specificity), and Kappa statistic between STRAT4 and IHC were assessed for each marker. The performance targets of this study were met if the lower 95 % 2-sided confidence limit for PPA and NPA exceeded the prospectively defined thresholds for each of the three targets: ER (PPA ≥80 %, NPA ≥80 %), PR (PPA ≥70 %, NPA ≥65 %), Ki67 (PPA ≥65 %, NPA ≥65 %) [8].

The secondary objective was to evaluate the concordance of STRAT4 and IHC in post-treatment surgical samples and the association of both assays with oncologic outcome parameters such as residual cancer burden (RCB), time to distant recurrence (DR), and overall survival (OS). RCB was analyzed by logistic regression models and time to DR as well as OS were analyzed by Cox models. To visualize absolute risk over time, Kaplan Meier survival curves were created.

Categorical baseline data according to treatment arms were compared in univariate analysis using the Chi-square or Fisher's exact test depending on the expected cell frequencies. Continuous baseline data were compared using Wilcoxon test. All reported P-values are from two-sided tests. All results with P-values <0.05 were considered statistically significant. Statistical analyses were performed by members of the biostatistics group at ABCSG using statistical analysis system (SAS) software (SAS® version 9.3 or higher). This study follows the Reporting Recommendations for Tumor Marker Prognostic Studies (REMARK) [11].

## 3 Results

### 3.1 Patient characteristics

Diagnostic core biopsy samples were available from 354 of the 400 patients enrolled in the ABCSG Trial 34 (272 patients from the NaCT cohort and 82 patients from the NET cohort) and post neoadjuvant treatment surgical samples from 249 patients (171 patients from the NaCT cohort and 78 patients from the NET cohort). This corresponds to 88.5 % of all available core biopsy samples from enrolled patients. Clinical and laboratory parameters of all patients stratified by treatment are shown in Table 1. None of the biopsy samples required macro-dissection, while 147 of 249 (59 %) of the surgical samples had a tumor cell content of less than 30 % and were macro-dissected.

**Table 1:** Baseline characteristics stratified by treatment.

Variable	NaCT (n = 272)	NET (n = 82)	Total (n = 354)
Age, mean (SD)	49.0 (10.9)	66.6 (7.2)	53.1 (12.6)
Menopausal status			
Premenopausal	164 (60.3 %)	1 (1.2 %)	165 (46.6 %)
Perimenopausal	9 (3.3 %)	0	9 (2.5 %)
Postmenopausal	95 (34.9 %)	81 (98.8 %)	176 (49.7 %)
Missing	4 (1.5 %)	0	4 (1.1 %)
cT-Stage			
T1	76 (27.9 %)	38 (46.3 %)	114 (32.2 %)
T2	168 (61.8 %)	40 (48.8 %)	208 (58.8 %)
T3	25 (9.2 %)	3 (3.7 %)	28 (7.9 %)
T4	3 (1.1 %)	0	3 (0.8 %)
Missing	0	1 (1.2 %)	1 (0.3 %)
cN-Stage			
N0	159 (58.5 %)	67 (81.7 %)	226 (63.8 %)
N1	101 (37.1 %)	15 (18.3 %)	116 (32.8 %)
N2	2 (0.7 %)	0	2 (0.6 %)
N3	4 (1.5 %)	0	4 (1.1 %)
Missing	6 (2.2 %)	0	6 (1.7 %)
Grading			
G1	2 (0.7 %)	12 (14.6 %)	14 (4.0 %)
G2	68 (25.0 %)	59 (72.0 %)	127 (35.9 %)
G3	186 (68.4 %)	4 (4.9 %)	190 (53.7 %)
GX or missing	16 (5.9 %)	7 (8.5 %)	23 (6.5 %)
ER			
<1 %	118 (43.4 %)	0	118 (33.3 %)
≥1 %	152 (55.9 %)	82 (100.0 %)	234 (66.1 %)
Missing	2 (0.7 %)	0	2 (0.6 %)
PR			
<1 %	115 (42.3 %)	1 (1.2 %)	116 (32.8 %)
≥1 %	154 (56.6 %)	80 (97.6 %)	234 (66.1 %)
Missing	3 (1.1 %)	1 (1.2 %)	4 (1.1 %)
HER2			
Negative	270 (99.3 %)	81 (98.8 %)	351 (99.2 %)
Positive	2 (0.7 %)	0	2 (0.6 %)
Missing	0	1 (1.2 %)	1 (0.3 %)
Ki67			
≤5 %	5 (1.8 %)	3 (3.7 %)	8 (2.3 %)
6–29 %	36 (13.2 %)	58 (70.7 %)	94 (26.6 %)
≥30 %	230 (84.6 %)	21 (25.6 %)	251 (70.9 %)
Missing	1 (0.4 %)	0	1 (0.3 %)
*ESR1*			
Negative	138 (50.7 %)	1 (1.2 %)	139 (39.3 %)
Positive	132 (48.5 %)	81 (98.8 %)	213 (60.2 %)
Invalid	2 (0.7 %)	0	2 (0.6 %)
*PGR*			
Negative	140 (51.5 %)	10 (12.2 %)	150 (42.4 %)
Positive	125 (46.0 %)	70 (85.4 %)	195 (55.1 %)
Invalid	2 (0.7 %)	0	2 (0.6 %)
Indeterminate	5 (1.8 %)	2 (2.4 %)	7 (2.0 %)
*ERBB2*			
Negative	266 (97.8 %)	79 (96.3 %)	345 (97.5 %)
Positive	4 (1.5 %)	3 (3.7 %)	7 (2.0 %)
Invalid	2 (0.7 %)	0	2 (0.6 %)
*MKi67*			
Low	32 (11.8 %)	54 (65.9 %)	86 (24.3 %)
High	232 (85.3 %)	26 (31.7 %)	258 (72.9 %)
Invalid	2 (0.7 %)	0	2 (0.6 %)
Indeterminate	6 (2.2 %)	2 (2.4 %)	8 (2.3 %)

SD = standard deviation; NaCT = neoadjuvant chemotherapty; NET = neoadjuvant endocrine treament.

### 3.2 Concordance between STRAT4 mRNA measurements and IHC

The primary objective of our study was to demonstrate concordance between STRAT4 mRNA measurement and central IHC results tested on diagnostic core biopsy samples from all available patients. Table 2 contains 2 by 2 tables with STRAT4 mRNA measurements of *ESR1*, *PGR*, *ERBB2*, and *MKi67* and central reference laboratory IHC (and ISH for HER2 IHC 2+ cases) in ABCSG Trial 34 core biopsy samples. Cases with indeterminate or invalid STRAT4 results were excluded from the concordance analyses (Table 1).

**Table 2:** Concordance between STRAT4 and IHC.

Variable	IHC^+^/STRAT4^+^	IHC^−^/STRAT4^+^	IHC^+^/STRAT4^-^	IHC^−^/STRAT4^-^	Concordance OPA % (95 %CI)	Sensitivity PPA % (95 %CI)	Specificity NPA % (95 %CI)	Kappa statistic (95 %CI)
Diagnostic core biopsies								
*ESR1*/ER_<1 % vs. ≥1 %_	212	1	21	117	93.7 (90.7–95.8)	91.0 (86.6–94.0)	99.2 (95.4–99.9)	0.87 (0.81–0.92)
*ESR1*/ER_<10 % vs. ≥10 %_	210	3	6	132	97.4 (95.2–98.6)	97.2 (94.1–98.7)	97.8 (93.7–99.2)	0.95 (0.91–0.98)
*PGR*/PR_<1 % vs. ≥1 %_	179	16	51	98	80.5 (76.0–84.4)	77.8 (72.0–82.7)	86.0 (78.4–91.2)	0.59 (0.51–0.68)
*PGR*/PR_<10 % vs. ≥10 %_	171	24	22	127	86.6 (82.6–89.8)	88.6 (83.3–92.4)	84.1 (77.4–89.1)	0.73 (0.66–0.80)
*ERBB2*/HER2	1	6	1	343	–	–	–	–
*MK67*/Ki67_≤5 % vs. ≥30 %_	232	0	15	7	94.1 (90.5–96.4)	93.9 (90.2–96.3)	100.0 (72.1–100.0)	0.46 (0.24–0.68)
*MK67*/Ki67_≤20 % vs. >20 %_	232	26	15	71	88.1 (84.2–91.1)	93.9 (90.2–96.3)	73.2 (63.6–81.0)	0.70 (0.61–0.78)
**Post-treatment surgical samples**								
*ESR1*/ER_<1 % vs. ≥1 %_	170	15	5	56	91.9 (87.8–94.7)	97.1 (93.5–98.8)	78.9 (68.0–86.8)	0.79 (0.71–0.88)
*ESR1*/ER_≥<10 % vs. ≥10 %_	169	16	4	57	91.9 (87.8–94.7)	97.7 (94.2–99.1)	78.1 (67.3–86.0)	0.80 (0.71–0.88)
*PGR*/PR_<1 % vs. ≥1 %_	121	45	14	62	75.6 (69.8–80.6)	89.6 (83.3–93.7)	57.9 (48.5–66.9)	0.49 (0.38–0.60)
*PGR*/PR_<10 % vs. ≥10 %_	101	65	6	70	70.7 (64.6–76.0)	94.4 (88.3–97.4)	51.9 (43.5–60.1)	0.44 (0.34–0.54)
*ERBB2*/HER2	0	1	3	224	–	–	–	–
*MK67*/Ki67_≤5 % vs. ≥30 %_	64	4	10	113	92.7 (88.1–95.6)	86.5 (76.9–92.5)	96.6 (91.5–98.7)	0.84 (0.76–0.92)
*MK67*/Ki67_≤20 % vs. >20 %_	64	20	10	144	87.4 (82.6–91.0)	86.5 (76.9–92.5)	87.8 (81.9–92.0)	0.72 (0.62–0.81)

**Fig. 1:**
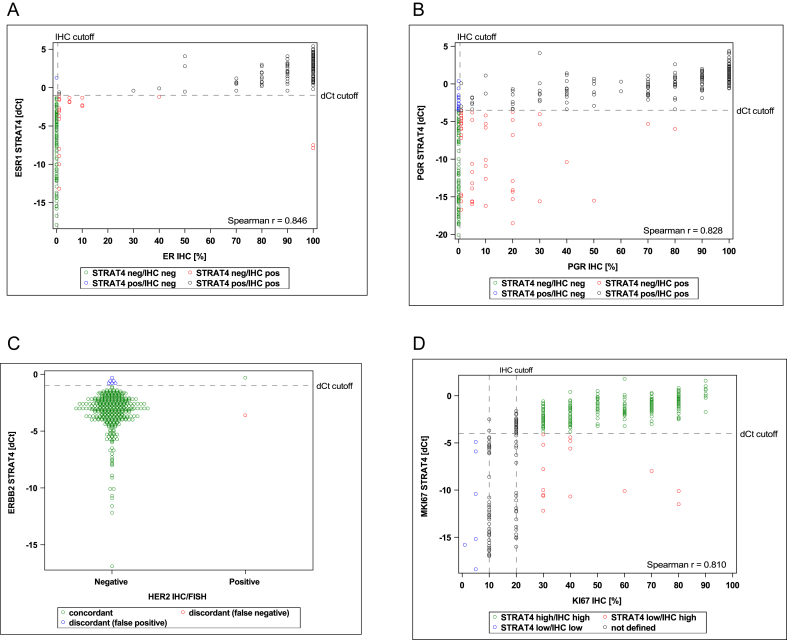
Scatter plots of STRAT4 mRNA results and central IHC in ABCSG Trial 34.

The primary objective for ER was met. Two cases (0.6 %) with invalid STRAT4 *ESR1* results were excluded from the concordance analysis (Table 1, Table 2). The lower limits of the 95 % confidence intervals (CIs) for PPA and NPA between STRAT4 *ESR1* mRNA measurements and ER central IHC (cutoff ≥1 %) in 351 cases were 86.6 % and 95.4 %, respectively (concordance 90.7 %) (Table 2). *ESR1* ΔCt values were plotted against percent positive ER staining treated as continuous variables and for the same samples. These data show the high correlation between STRAT4 *ESR1* mRNA and central ER IHC (Fig. 1A). Similar results were observed when the cutoff ≥10 % was applied for ER IHC (lower limits of the 95 % CIs for PPA: 94.1 % and NPA: 93.7 %, respectively) (Table 2). In surgical samples obtained after neoadjuvant treatment, the lower limits of the 95 % CIs for PPA and NPA between STRAT4 *ESR1* mRNA measurements and ER central IHC (cutoff ≥1 %) in 246 cases were 93.5 % and 68.0 %, respectively (concordance 87.8 %) (Table 2).

The primary objective for PR was met. Seven (2.0 %) cases with indeterminate and 2 (0.6 %) cases with invalid STRAT4 *PGR* results were excluded from the concordance analysis (Table 2). The lower limits of the 95 % CIs for PPA and NPA of the STRAT4 *PGR* mRNA results compared with PR central IHC (cutoff ≥1 %) in 344 cases were 72.0 % and 78.4 %, respectively (concordance 76.0 %) (Table 2). Scatterplots of STRAT4 *PGR* mRNA compared with PR protein suggest a positive correlation (Fig. 1B). Using a cut-off of ≥10 % for PR IHC, the lower limits of the 95 % CIs were 83.3 % for PPA and 77.4 %for NPA (Table 2). In surgical samples, the lower limits of the 95 % CIs for PPA and NPA between STRAT4 *PGR* mRNA measurements and PR central IHC (cutoff ≥1 %) in 242 cases were 83.3 % and 48.5 %, respectively (concordance 69.8 %) (Table 2).

Only patients with HER2-negative tumors were included in ABCSG Trial 34, therefore, no results regarding HER2 concordance were shown due to the small number of HER2-positive cases. Most IHC HER2-negative cases were also negative by STRAT4 analysis (Table 2). A comparison of STRAT4 *ERBB2* ΔCt values and IHC results is shown in a scatterplot (Fig. 1C).

The primary objective for Ki67 was met. For *MKi67*/Ki67 concordance, 8 (2.3 %) samples with indeterminate STRAT4 results, 2 (0.6 %) samples with invalid STRAT4 *MKi67* results and 94 samples with intermediate (10–20 %) Ki67 protein expression were excluded from the primary concordance analysis (Table 2). The lower limits of the 95 % CIs for PPA and NPA between STRAT4 *MKi6*7 mRNA results and Ki67 protein expression by IHC in 254 cases were 90.2 % and 72.1 %, respectively (concordance 90.5 %) (Table 2). A scatterplot of MKi67 ΔCt values with Ki67 IHC results is shown in Fig. 1D. In surgical samples, the lower limits of the 95 % CIs for PPA and NPA between STRAT4 *MKI6*7 mRNA measurements and Ki67 central IHC in 191 cases were 76.9 % and 91.5 %, respectively (concordance 88.1 %) (Table 2).

### 3.3 Comparison of STRAT4 biomarker expression in biopsies and surgical samples

A comparison of *ESR1/*ER, *PGR/*PR, *ERBB2/*HER2, and *MKI67/*Ki67 expression in diagnostic core biopsies and surgical samples after neoadjuvant treatment yielded very similar STRAT4 and IHC results (Table S3). In 221 of 246 (89.8 %) samples, *ESR1* expression did not change between pretreatment biopsy specimens and surgical samples and in 232 of 245 (94.7 %) samples ER expression remained unchanged. Similar results were found between STRAT4 mRNA measurements and IHC in biopsy specimens compared to surgical samples for *PGR* and PR expression (178 of 235 [75.7 %] and 180 of 243 [74.1 %]), *ERBB2* and HER2 expression (239 of 246 [97.2 %] and 223 of 226 [98.7 %]), and *MKI67* and Ki67 expression (144 of 233 [61.8 %] and 75 of 125 [60.0 %]) (Table S3).

### 3.4 Correlation of STRAT4 with response to neoadjuvant therapy

Response to neoadjuvant therapy was measured using the Residual Cancer Burden (RCB). A RCB score was available for 330 patients of whom 64 had RCB0 (pCR) (251 patients from the NaCT cohort and 79 patients from the NET cohort). The association between STRAT4 and RCB was evaluated separately in the NaCT and NET cohorts.

In the NaCT cohort, continuous *ESR1*, *PGR*, and *MKi67* ΔCt values were significantly correlated with RCB. Tumors with lower *ESR1*, *PGR*, or higher *MKi67* ΔCt values had a higher probability of achieving RCB 0/1 (Table 3). In the NET cohort, tumors with lower *ERBB2* or lower *MKi67* ΔCt values had a higher probability of achieving RCB 0/1 (Table 3). Similar results were shown for continuous ER, PR, and Ki67 protein expression and RCB (Table 3). In the NaCT cohort, tumors with lower ER or PR expression as well as tumors with higher Ki67 expression had a higher probability of achieving RCB 0/1 (Table 3), whereas in the NET cohort, only tumors with lower Ki67 expression had a higher probability of achieving RCB 0/1 (Table 3). Due to the extremely small number of HER2-positive cases, HER2 was excluded from these analyses.

**Table 3:** Univariate logistic regression models – continuous variables.

Arm	Variable	N	Events	Odds ratio (95 % CI)	P-value
NaCT	*ESR1*	251	92	0.93 (0.89–0.98)	0.007
	*PGR*	251	92	0.91 (0.87–0.95)	<0.0001
	*ERBB2*	251	92	0.94 (0.82–1.08)	0.39
	*MKI67*	251	92	1.23 (1.08–2.05)	0.002
NET	*ESR1*	79	15	0.76 (0.54–1.08)	0.12
	*PGR*	79	15	1.01 (0.89–1.14)	0.90
	*ERBB2*	79	15	0.58 (0.36–0.93)	0.02
	*MKI67*	79	15	0.89 (0.79–1.00)	0.05
NaCT^b^	ER^b^	250	91	0.88 (0.83–0.93)	<0.0001
	PR^b^	249	91	0.81 (0.75–0.89)	<0.0001
	KI67^b^	251	92	1.35 (1.19–1.53)	<0.0001
NET^a^	ER^b^	79	15	1.05 (0.41–2.72)	0.91
	PR^b^	78	14	1.22 (0.95–1.57)	0.11
	KI67^b^	79	15	0.32 (0.12–0.83)	0.02

NaCT = neoadjuvant chemotherapy.

NET = neoadjuvant endocrine therapy.

a Due to the small number of HER2-positive cases, HER2 was excluded from these analyses.

b Odds ratio for an absolute 10 %-point increase.

### 3.5 Correlation of STRAT4 with time to distant recurrence and overall survival

We were able to retrospectively collect additional clinical follow-up data from 264 patients for whom a diagnostic core biopsy was available (213 from the NaCT cohort and 51 from the NET cohort) after study termination. A comparison of baseline characteristics between patients with follow-up data versus patients without follow-up data is shown in Table S1. At a median follow-up of 7.3 years from surgery and 7.9 years from randomization, 64 patients had developed distant recurrences (57 patients in the NaCT arm and 7 patients in the NET arm) and 68 patients had died (52 patients in the NaCT arm and 16 patients in the NET arm). Due to the small sample size and low event rate in the NET cohort, survival analyses were performed only in the NaCT cohort. For the same reason, HER2 was excluded from the survival analyses.

**Fig. 2:**
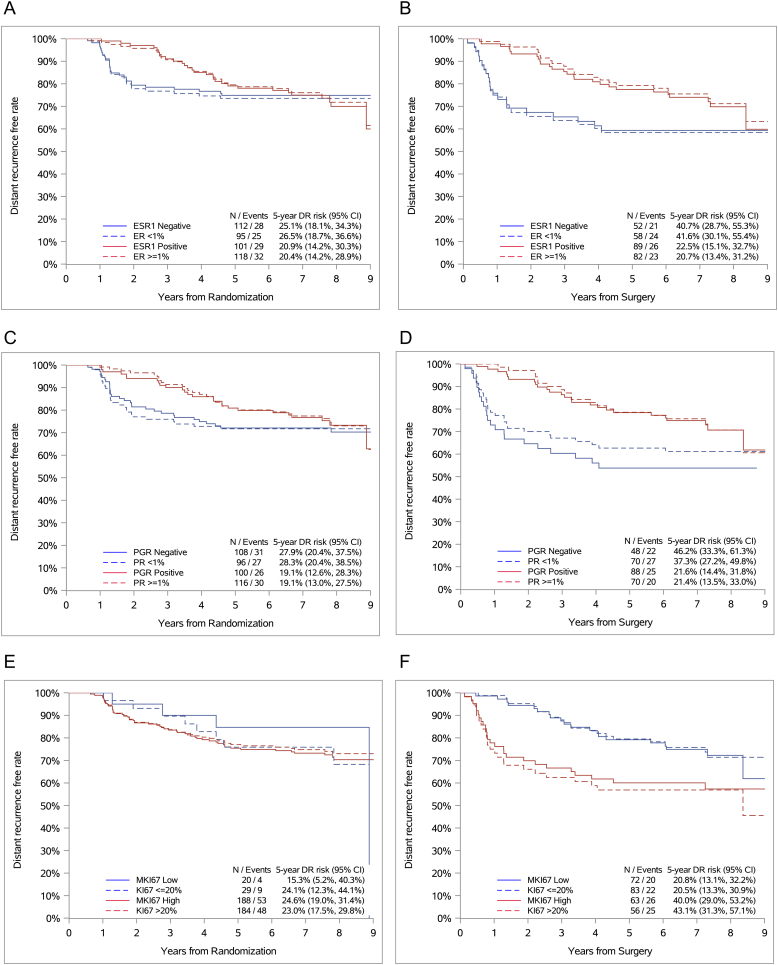
Kaplan-Meier plots for time to distant recurrence according to *ESR1*/ER, *PGR*/PR, and *MKi67*/Ki67 in diagnostic core biopsies (A, C, E) and surgical specimens (B, D, F).

In diagnostic core biopsies, univariate analyses showed that, with the exception of continuous *ESR1* (P = 0.03)*,* none of the continuous or categorical STRAT4 or IHC markers in the NaCT cohort were significantly associated with time to DR or OS from randomization (Table 4). Similar 5-year DR rates were observed for *ESR1*/ER, *PGR*/PR, and *MKi67*/Ki67 (Fig. 2A, C and 2E). Comparable results were obtained for 5-year OS rates (Figs. S1A, S1C, and S1E). In surgical samples, univariate analyses showed that continuous *ESR1* (P = 0.002) and *MKi67* (P = 0.0004) mRNA expression as well as Ki67 (P = 0.001) protein expression were significantly associated with time to distant recurrence from surgery (Table S2). Continuous *ESR1* (P < 0.0001), *PGR* (P = 0.005), *MKi67* (P < 0.0001), ER (P = 0.003), and Ki67 (P < 0.0001) were also significantly associated with overall survival from surgery (Table S2). Moreover, we found similar 5-year DR rates for *ESR1*/ER, *PGR*/PR, and *MKi67*/Ki67 (Fig. 2B, D, 2F). The mRNA expression of categorical *ESR1* (P = 0.008), *PGR* (P < 0.001), and *MKi67* (P = 0.001) as well as the categorical protein expression of ER (P < 0.001), PR (P = 0.008), and Ki67(P < 0.001) in surgical specimens were also significantly associated with overall survival (Table S2). Comparable 5-year OS rates were observed for *ESR1*/ER, *PGR*/PR, and *MKi67*/Ki67 (Figs. S1B, S1D, S1F).

**Table 4:** Univariable cox proportional hazard models of baseline markers in the NaCT cohort – time to distant recurrence or overall survival from randomization.

Continuous variables								
Variable	Time to distant recurrence				Overall survival			
	N	Events	HR (95 % CI)	P-value	N	Events	HR (95 % CI)	P-value
*ESR1*	213	57	0.97 (0.92–1.02)	0.24	213	52	0.95 (0.90–0.99)	0.03
*PGR*	213	57	0.99 (0.95–1.03)	0.74	213	52	0.97 (0.93–1.01)	0.21
*ERBB2*	213	57	1.01 (0.87–1.16)	0.94	213	52	0.99 (0.86–1.14)	0.86
*MKI67*	213	57	1.02 (0.93–1.11)	0.72	213	52	1.01 (0.92–1.10)	0.90
ER^a^	213	57	1.01 (0.96–1.07)	0.70	213	52	0.98 (0.93–1.04)	0.58
PR^a^	212	57	0.97 (0.90–1.03)	0.32	212	52	0.92 (0.85–1.00)	0.05
KI67^a^	213	57	0.92 (0.82–1.03)	0.14	213	52	0.98 (0.87–1.10)	0.71

**Categorical variables**								

**Variable**	**Time to distant recurrence**				**Overall survival**			
	**N**	**Events**	**HR (95 % CI)**	**P-value**	**N**	**Events**	**HR (95 % CI)**	**P-value**
*ESR1*	213	57	1.01 (0.60–1.71)	0.96	213	52	0.79 (0.46–1.36)	0.39
*PGR*	208	57	0.81 (0.48–1.36)	0.43	208	52	0.67 (0.39–1.17)	0.16
*MKI67*	208	57	1.51 (0.55–4.18)	0.43	208	52	1.33 (0.48–3.69)	0.58
ER_<1 % vs. ≥1 %_	213	57	0.90 (0.53–1.52)	0.70	213	52	0.65 (0.38–1.12)	0.12
PR_<1 % vs. ≥1 %_	212	57	0.78 (0.47–1.32)	0.36	212	52	0.61 (0.35–1.05)	0.07
KI67_≤20 % vs. >20 %_	212	56	0.94 (0.44–1.99)	0.87	213	52	0.85 (0.40–1.81)	0.68

a HR for an absolute 10 %-point increase.

## 4 Discussion

The biomarkers ER, PR, HER2 and Ki67 assessed by IHC have prognostic and predictive value and are therefore essential in the therapeutic management of breast cancer [12]. Yet, up to 20 % of ER/PR testing may be incorrect due to pre-analytic variability, variable definitions of thresholds and varying criteria of test interpretation [5]. Consequently, there is a clear need for more standardized test strategies. In the present study we evaluated the concordance of the STRAT4 assay – that allows cartridge-based RT-qPCR detection of mRNA – to central IHC of ER, PR, HER2 and Ki67 using archived FFPE breast cancer tissue from the ABCSG Trial 34. This allowed us to evaluate the performance of the STRAT4 assay in diagnostic core biopsies and surgical resection specimens.

The primary objective, concordance between STRAT4 mRNA measurements with central reference laboratory IHC in diagnostic core biopsies, was met. We found high overall agreement between STRAT4 and IHC results for all four markers.

Only two diagnostic core biopsies of 354 evaluated samples had an invalid STRAT4 result, demonstrating the high robustness of the test which was also demonstrated previously [13]. The use of the ABCSG Trial 34 biomarker cohort furthermore allowed evaluation of the clinical relevance of the STRAT4 assay by correlating STRAT4 and IHC results with RCB and long-term outcome. RCB predicts disease recurrence and survival across all breast cancer subtypes [14]. It is a well-established outcome parameter to evaluate treatment efficacy in neoadjuvant trials and is used to tailor post-neoadjuvant treatment decisions to the patients’ oncologic risk [[15], [16], [17]]. In the present study, the STRAT4 markers *ESR1*, *PGR*, and *MKi67* were significantly correlated with RCB demonstrating the clinical relevance of the STRAT4 assay for predicting response to NaCT. Similar results were found for IHC markers. Furthermore, comparable 5-year DR and OS rates were found for STRAT4 markers and IHC markers.

Similar results on concordance and prognostic value were found when comparing the performance of STRAT4 with central IHC in FFPE breast cancer samples of the ABCSG Trial 6 [8]. In addition, good concordance of the STRAT4 assay with IHC was confirmed by others [7,13,[18], [19], [20], [21], [22], [23]]. MammaTyper®, another available mRNA assay, showed good concordance for ESR1/ER (Kappa: 0.83, PPA: 91.7 %, NPA: 92.9 %, OPA 92.1 %) and ERBB2/HER2 (Kappa: 0.85, PPA: 89.3 %, NPA: 95.5 %, OPA 93.1 %) as well as a moderate agreement between PGR/PR (Kappa: 0.53, PPA: 70.7 %, NPA: 82.9 %, OPA 76.3 %) and MKI67/Ki67 (Kappa: 0.46, PPA: 94.4 %, NPA: 60.0 %, OPA 92.1 %) [24]. Compared to MammaTyper, STRAT4 showed similar performance: good concordance for ESR1/ER (Kappa: 0.87, PPA: 91.0 %, NPA: 99.2 %, OPA 93.7 %) and moderate agreement between PGR/PR (Kappa: 0.59, PPA: 77.8 %, NPA: 86.0 %, OPA 80.5 %) and MKI67/Ki67 (Kappa: 0.70, PPA: 93.9 %, NPA: 73.2 %, OPA 88.1 %).

In addition to analytical performance comparable to IHC, the short turnaround time of the assay, including off-board lysate preparation and assay run time, of less than 2 h, as well as the lower costs compared to IHC plus HER2 ISH, were also recognized [25,26]. Additional advantages of the STRAT4 assay include its ease of use and the global availability of GX systems, even in many low-to-middle income countries where IHC is either unaffordable and/or not feasible due to a lack of resources (few pathologists, qualified technical staff). Therefore, STRAT4 can be used in low resource settings where many other Xpert tests (e.g., HIV, TB, CT/NG, SARS-CoV-2, etc.) are already in use. However, the STRAT4 assay also has limitations, including its poorer performance in invasive carcinomas with extensive intraductal component, DCIS with microinvasion and minimal residual disease following neoadjuvant treatment. This requires quality assurance of the sample by a pathologist and the exclusion of larger amounts of adjacent normal breast tissue and precursor lesions such as atypical ductal hyperplasia. Furthermore, in case of “borderline” or indeterminate results due to analytic problems or in case of low or heterogeneous biomarker expression, IHC has to be complemented.

The strengths of our study are a well-documented patient cohort, the use of analytically validated assays, the blindness of laboratory staff to clinical data, and the availability of high-level central pathology reviews as the gold standard. Furthermore, diagnostic core biopsy samples from 88.5 % of patients and additional follow-up data from a prospective neoadjuvant randomized phase II trial were available. Also, all statistical analyses were performed according to a predefined statistical analysis plan.

Limitations of this work are the retrospective nature and the fact that our study did not include enough patients with HER2-amplified tumors, so that no valid conclusions can be drawn for this patient cohort.

In summary, this study adds to the body of evidence of the comparable analytical performance of STRAT4 to IHC in diagnostic core biopsies and surgical samples. Our results suggest that STRAT4 is a promising, standardized, cost- and time efficient tool for biomarker analysis in breast cancer, and might provide a feasible alternative to IHC, especially in low-resource settings.

## CRediT authorship contribution statement

**Stephanie Kacerovsky-Strobl:** Writing – review & editing, Writing – original draft, Investigation. **Christine Deutschmann:** Writing – review & editing, Writing – original draft, Investigation. **Dominik Hlauschek:** Writing – review & editing, Writing – original draft, Methodology, Investigation, Formal analysis, Conceptualization. **Zsuzsanna Bago-Horvath:** Writing – review & editing, Writing – original draft, Resources, Investigation. **Christian F. Singer:** Writing – review & editing, Writing – original draft, Resources, Investigation. **Rupert Bartsch:** Writing – review & editing, Writing – original draft, Resources, Investigation. **Richard Greil:** Writing – review & editing, Writing – original draft, Resources, Investigation. **Karl Sotlar:** Writing – review & editing, Writing – original draft, Resources, Investigation. **Gabriel Rinnerthaler:** Writing – review & editing, Writing – original draft, Resources, Investigation. **Edgar Petru:** Writing – review & editing, Writing – original draft, Resources, Investigation. **Sigurd F. Lax:** Writing – review & editing, Writing – original draft, Resources, Investigation. **Daniel Egle:** Writing – review & editing, Writing – original draft, Resources, Investigation. **Angelika Pichler:** Writing – review & editing, Writing – original draft, Resources, Investigation. **Koppány Bodó:** Writing – review & editing, Writing – original draft, Resources, Investigation. **Andreas L. Petzer:** Writing – review & editing, Writing – original draft, Resources, Investigation. **Farid Moinfar:** Writing – review & editing, Writing – original draft, Resources, Investigation. **Jodi Weidler:** Writing – review & editing, Writing – original draft, Validation, Resources, Project administration, Methodology, Investigation, Funding acquisition, Formal analysis, Data curation, Conceptualization. **Michael Bates:** Writing – review & editing, Writing – original draft, Validation, Resources, Project administration, Methodology, Investigation, Funding acquisition, Formal analysis, Data curation, Conceptualization. **Peter Dubsky:** Writing – review & editing, Writing – original draft, Validation, Resources, Project administration, Methodology, Investigation, Funding acquisition, Formal analysis, Data curation, Conceptualization. **Michael Gnant:** Writing – review & editing, Writing – original draft, Resources, Investigation. **Martin Filipits:** Writing – review & editing, Writing – original draft, Validation, Supervision, Resources, Project administration, Methodology, Investigation, Funding acquisition, Formal analysis, Data curation, Conceptualization.

## Funding

This work was funded by 10.13039/100017037Cepheid.

## Declaration of competing interest

S.K.S has received travel grants from Roche, Novartis and AstraZeneca and speakers honoraria from AstraZeneca. C.D. has received travel grants from Roche, Novartis, AstraZeneca and DaiichiSankyo; consulting/advisory fees from Novartis and speakers honoraria from AstraZeneca and Daiichi Sankyo. D.H. is employed at ABCSG and ABCSG has received funding for the study from Cepheid. Z. B.-H. has received consulting/advisory fees and speaker honoraria from Gilead, MSD, Stemline, Daiichi Sankyo and Astra Zeneca. C.F.S. reports disclosures caused by paid consultancies (AstraZeneca, Novartis, Gilad) and research grants (Novartis, Amgen, Gilead, Daiichi Sankyo, AstraZeneca). R.B. has received personal fees/travel support from Amgen, Astra-Zeneca, BMS, Daichi, Eisai, Eli-Lilly, Gilead, Gruenenthal, MSD, MedMedia, Novartis, Pfizer, Pierre-Fabre, Roche, Seagen, Stemline. G.R. reports disclosures caused by consulting fees (Amgen, AstraZeneca, Daiichi Sankyo, Eli Lilly, Gilead, MSD, Novartis, Pfizer, Pierre Fabre, Roche, Stemline), payment/honoraria (Amgen, AstraZeneca, Daiichi Sankyo, Eli Lilly, Gilead, MSD, Novartis, Pfizer, Roche, Seagen, Stemline, BMS), and travel/accommodations/expenses (Amgen, Daiichi Sankyo, Eli Lilly, Gilead, Merck, Pfizer, Roche). S.F.L. has received fees for lectures and participation in advisory boards by Astra-Zeneca, Novartis, PharmaMar, Daiichi Sankyo, Biocartis, Merck Sharp & Dome (MSD) and GlaxoSmithKline (GSK). D.E. reports disclosures caused by honoraria (Roche, MSD, Novartis, AstraZeneca, Lilly, Gilead, Daiichii-Sankyo, Pfizer, Seagen), paid expert testimony (Gilead, AstraZeneca) and research grants/other funding (Sirius Medical) all outside the submitted work. A.P. has received honoraria and fees for participation in advisory boards by Novartis, Amgen, Celgene-BMS, Sandoz, Janssen, Astra Zeneca, Abbvie, Takeda, Sanofi, Kite-Gilead, Roche, Pfizer, Saegen, Daiichi Sankyo and travel support by Roche, Gilead, Daiichi Sankyo, Janssen, Eli Lilly, Pierre Fabre. J.W. and M.B. are employed by Cepheid, an operating company of Danaher, and have stock in Danaher. P.D. reports institutional support to Hirslanden Klink St. Anna for advisory/travel/accommodation from Roche, Astra Zeneca, Daiichi Sankyo, MSD. M.G. has received personal fees/travel support from Amgen, AstraZeneca, Bayer, DaiichiSankyo, EliLilly, EPG Health (IQVIA), Menarini-Stemline, MSD, Novartis, PierreFabre, Veracyte; an immediate family member is employed by Sandoz. M.F. has received honoraria from Astra Zeneca and Eli Lilly. All other authors declare no potential conflicts of interest.

## Footnotes

2 In vitro diagnostic medical device. Not available in all countries and in the U.S.
