# Supplementary material for: ESR1, PGR, ERBB2, and MKi67 mRNA expression in diagnostic core biopsies from breast cancer patients of the ABCSG Trial 34
Source: Breast. 2025 Oct 30;84:104633. doi: 10.1016/j.breast.2025.104633 (PMC12639300; doi:10.1016/j.breast.2025.104633)

**Table S1.** Baseline characteristics of patients with valid STRAT4 results in diagnostic core biopsies stratified by available follow-up data

| **Variable** | **Follow-up**  **(n = 264)** | **No follow-up**  **(n = 88)** | **Total**  **(n = 352)** |
| --- | --- | --- | --- |
| Treatment Arm | | | |
| NaCT | 213 (80.7%) | 57 (64.8%) | 270 (76.7%) |
| NET | 51 (19.3%) | 31 (35.2%) | 82 (23.3%) |
| Age | | | |
| Mean (SD) | 52.0 (12.2) | 56.5 (13.1) | 53.1 (12.5) |
| Menopausal status | | | |
| perimenopausal | 7 (2.7%) | 2 (2.3%) | 9 (2.6%) |
| postmenopausal | 123 (46.6%) | 52 (59.1%) | 175 (49.7%) |
| premenopausal | 131 (49.6%) | 33 (37.5%) | 164 (46.6%) |
| missing | 3 (1.1%) | 1 (1.1%) | 4 (1.1%) |
| Tumor size (cm) | | | |
| Mean (SD) | 3.0 (1.6) | 2.6 (1.3) | 2.9 (1.6) |
| cN-Stage | | | |
| N0 | 171 (64.8%) | 53 (60.2%) | 224 (63.6%) |
| N1 | 86 (32.6%) | 30 (34.1%) | 116 (33.0%) |
| N2 | 2 (0.8%) | 0 | 2 (0.6%) |
| N3 | 2 (0.8%) | 2 (2.3%) | 4 (1.1%) |
| Missing | 3 (1.1%) | 3 (3.4%) | 6 (1.7%) |
| Grading | | | |
| G1 | 8 (3.0%) | 6 (6.8%) | 14 (4.0%) |
| G2 | 83 (31.4%) | 43 (48.9%) | 126 (35.8%) |
| G3 | 152 (57.6%) | 37 (42.0%) | 189 (53.7%) |
| Gx or missing | 21 (8.0%) | 2 (2.3%) | 23 (6.5%) |
| ER | | | |
| <1% | 95 (36.0%) | 23 (26.1%) | 118 (33.5%) |
| ≥ 1% | 169 (64.0%) | 64 (72.7%) | 233 (66.2%) |
| missing | 0 | 1 (1.1%) | 1 (0.3%) |

**Table S1.** continued

| PR | | | |
| --- | --- | --- | --- |
| <1% | 97 (36.7%) | 19 (21.6%) | 116 (33.0%) |
| ≥ 1% | 165 (62.5%) | 68 (77.3%) | 233 (66.2%) |
| missing | 2 (0.8%) | 1 (1.1%) | 3 (0.9%) |
| HER2 | | | |
| negative | 262 (99.2%) | 87 (98.9%) | 349 (99.1%) |
| positive | 2 (0.8%) | 0 | 2 (0.6%) |
| Missing | 0 | 1 (1.1%) | 1 (0.3%) |
| Ki67 | | | |
| ≤5% | 6 (2.3%) | 2 (2.3%) | 8 (2.3%) |
| 6-29% | 56 (21.2%) | 37 (42.0%) | 93 (26.4%) |
| ≥30% | 202 (76.5%) | 49 (55.7%) | 251 (71.3%) |
| *ESR1* | | | |
| positive | 151 (57.2%) | 62 (70.5%) | 213 (60.5%) |
| negative | 113 (42.8%) | 26 (29.5%) | 139 (39.5%) |
| *PGR* | | | |
| positive | 142 (53.8%) | 53 (60.2%) | 195 (55.4%) |
| Negative | 115 (43.6%) | 35 (39.8%) | 150 (42.6%) |
| indeterminate | 7 (2.7%) | 0 | 7 (2.0%) |
| *ERBB2* | | | |
| negative | 258 (97.7%) | 87 (98.9%) | 345 (98.0%) |
| positive | 6 (2.3%) | 1 (1.1%) | 7 (2.0%) |
| *MKi67* | | | |
| High | 209 (79.2%) | 49 (55.7%) | 258 (73.3%) |
| Low | 48 (18.2%) | 38 (43.2%) | 86 (24.4%) |
| Indeterminate | 7 (2.7%) | 1 (1.1%) | 8 (2.3%) |

NaCT = neoadjuvant chemotherapy; NET = neoadjuvant endocrine therapy

**Table S2.** Univariable cox proportional hazard models of surgical markers in the NaCT cohort – time to distant recurrence or overall survival from surgery

**Continuous variables**

|  |  |  | **Time to distant recurrence** |  |  |  | **Overall survival** |  |
| --- | --- | --- | --- | --- | --- | --- | --- | --- |
| **Variable** | **N** | **Events** | **HR (95% CI)** | **P-value** | **N** | **Events** | **HR (95% CI)** | **P-value** |
| *ESR1* | 141 | 47 | 0.88 (0.82 - 0.96) | 0.002 | 141 | 43 | 0.86 (0.80 - 0.93) | <.0001 |
| *PGR* | 141 | 47 | 0.94 (0.88 - 1.00) | 0.07 | 141 | 43 | 0.91 (0.85 - 0.97) | 0.005 |
| *ERBB2* | 141 | 47 | 1.02 (0.87 - 1.19) | 0.84 | 141 | 43 | 1.00 (0.85 - 1.17) | 0.98 |
| *MKI67* | 141 | 47 | 1.13 (1.06 - 1.21) | 0.0004 | 141 | 43 | 1.19 (1.10 - 1.29) | <.0001 |
|  |  |  |  |  |  |  |  |  |
| ER* | 140 | 47 | 0.94 (0.89 - 1.00) | 0.06 | 140 | 43 | 0.91 (0.85 - 0.97) | 0.003 |
| PR* | 140 | 47 | 0.97 (0.90 - 1.06) | 0.53 | 140 | 43 | 0.93 (0.85 - 1.02) | 0.13 |
| KI67* | 139 | 47 | 1.17 (1.06 - 1.28) | 0.001 | 139 | 43 | 1.23 (1.12 - 1.35) | <.0001 |

*HR for an absolute 10%-point increase.

**Categorical variables**

|  |  |  | **Time to distant recurrence** |  |  |  | **Overall survival** |  |
| --- | --- | --- | --- | --- | --- | --- | --- | --- |
| **Variable** | **N** | **Events** | **HR (95% CI)** | **P-value** | **N** | **Events** | **HR (95% CI)** | **P-value** |
| *ESR1* | 141 | 47 | 0.58 (0.32 - 1.03) | 0.06 | 141 | 43 | 0.45 (0.25 - 0.81) | 0.008 |
| *PGR* | 136 | 47 | 0.46 (0.26 - 0.82) | 0.008 | 136 | 43 | 0.36 (0.20 - 0.66) | <0.001 |
| *MKI67* | 135 | 46 | 1.88 (1.05 - 3.37) | 0.03 | 135 | 42 | 2.91 (1.53 - 5.54) | 0.001 |
|  |  |  |  |  |  |  |  |  |
| ER_<1% vs. ≥1%_ | 140 | 47 | 0.51 (0.29 - 0.90) | 0.02 | 140 | 43 | 0.35 (0.19 - 0.65) | <0.001 |
| PR_<1% vs. ≥1%_ | 140 | 47 | 0.58 (0.33 - 1.04) | 0.07 | 140 | 43 | 0.43 (0.23 - 0.80) | 0.008 |
| KI67_≤20% vs. >20%_ | 139 | 47 | 2.21 (1.25 - 3.93) | 0.007 | 139 | 43 | 2.95 (1.60 - 5.44) | <0.001 |

**Table S3.** Comparison of STRAT4 biomarker expression in biopsies and surgical samples

STRAT4 *ESR1* ER IHC

Surgery Surgery

Baseline Negative Positive Baseline <1% ≥1%

Negative 54 18 <1% 59 2

Positive 7 167 ≥1% 11 173

STRAT4 *PGR* PR IHC

Surgery Surgery

Baseline Negative Positive Baseline <1% ≥1%

Negative 50 34 <1% 52 8

Positive 23 128 ≥1% 55 128

STRAT4 *ERBB2* HER2 IHC

Surgery Surgery

Baseline Negative Positive Baseline Negative Positive

Negative 239 1 Negative 223 3

Positive 6 0 Positive 0 0

STRAT4 *MKI67* Ki67 IHC

Surgery Surgery

Baseline Low High Baseline ≤5% ≥30%

Low 68 6 ≤5% 5 1

High 83 76 ≥30% 49 70

**Figure S1.** Kaplan-Meier plots for overall survival according to *ESR1*/ER, *PGR*/PR, and *MKi67*/Ki67 in diagnostic core biopsies (A, C, E) and surgical specimens (B, D, F).

A B


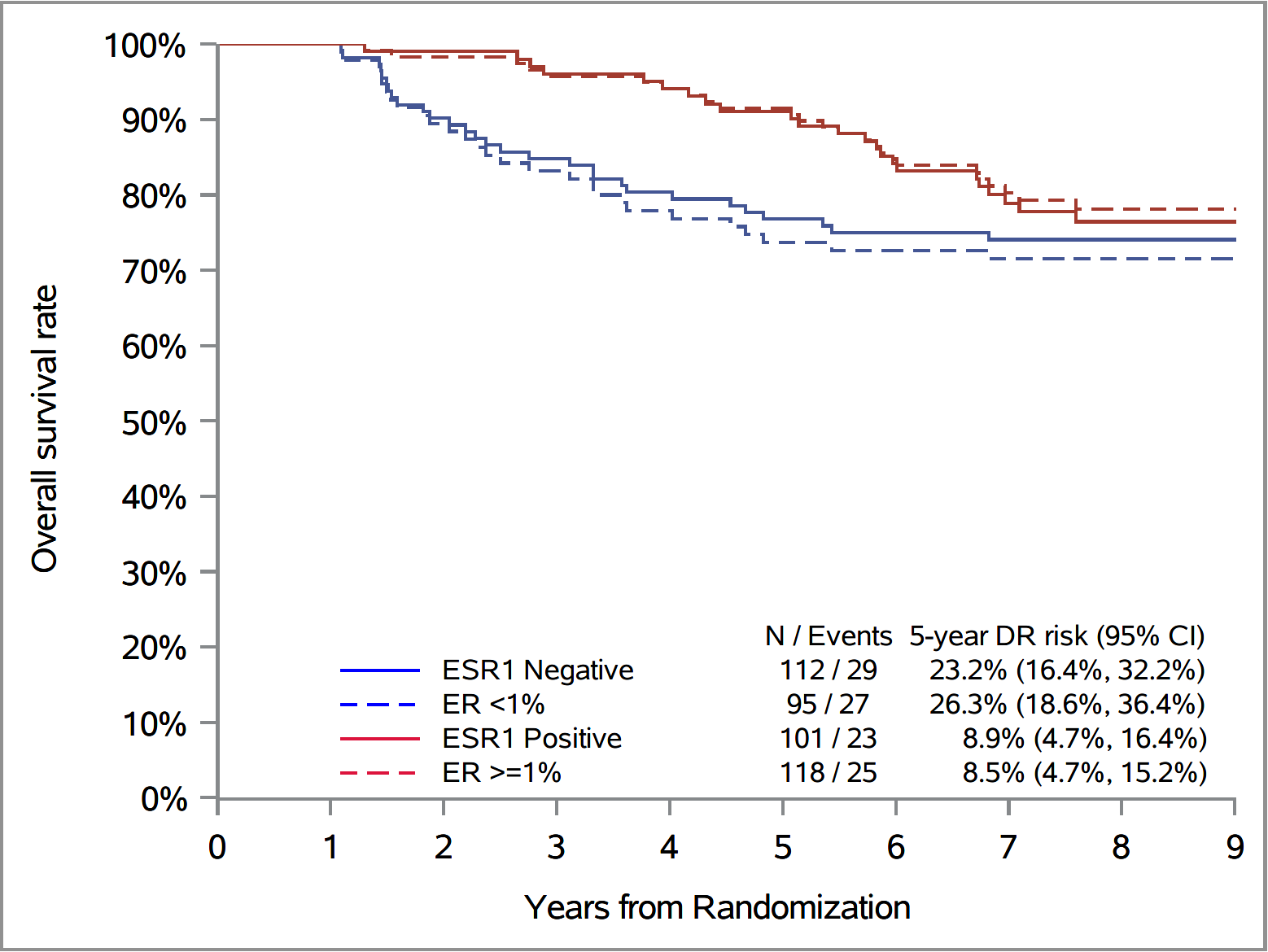

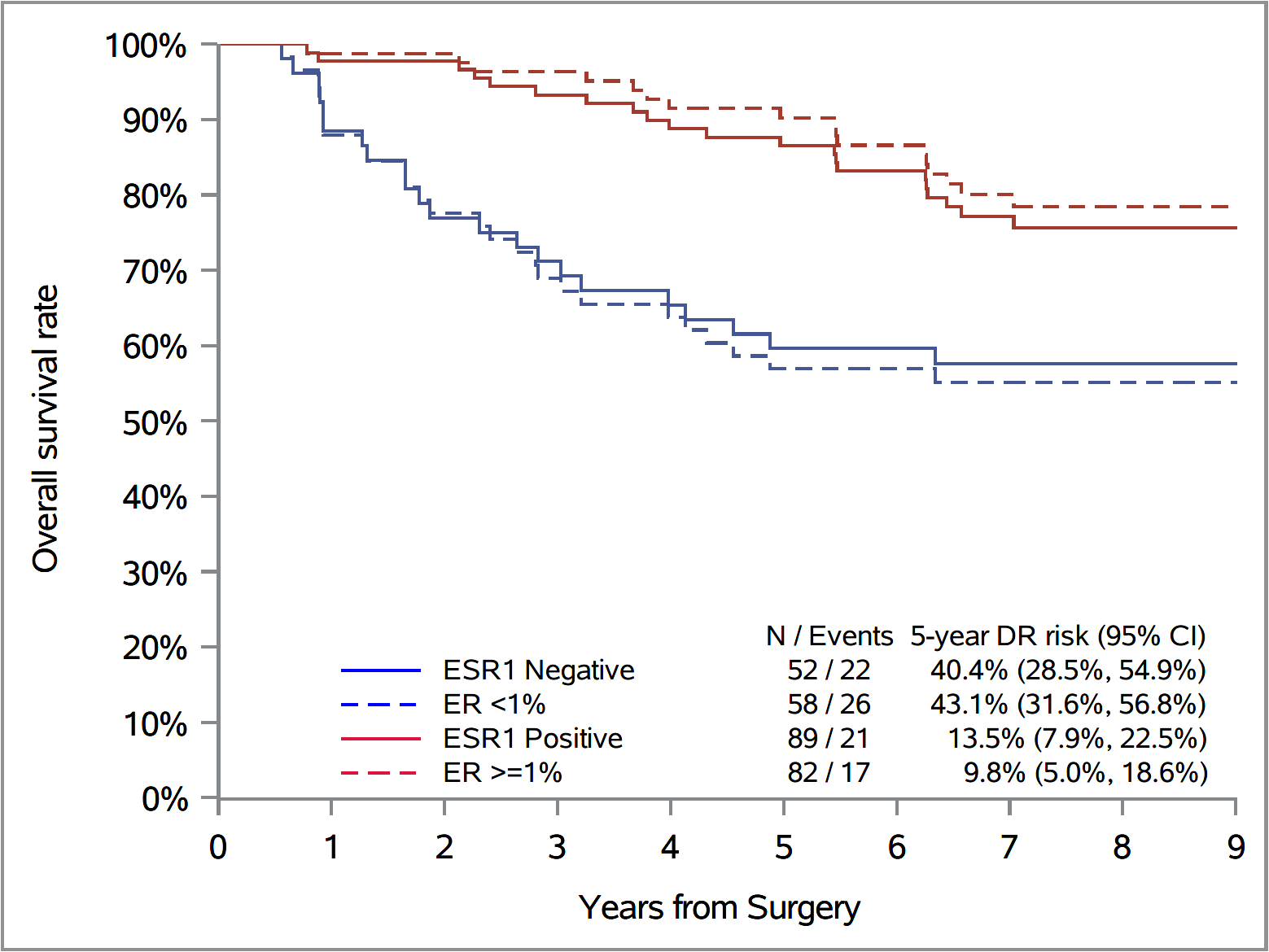


C D


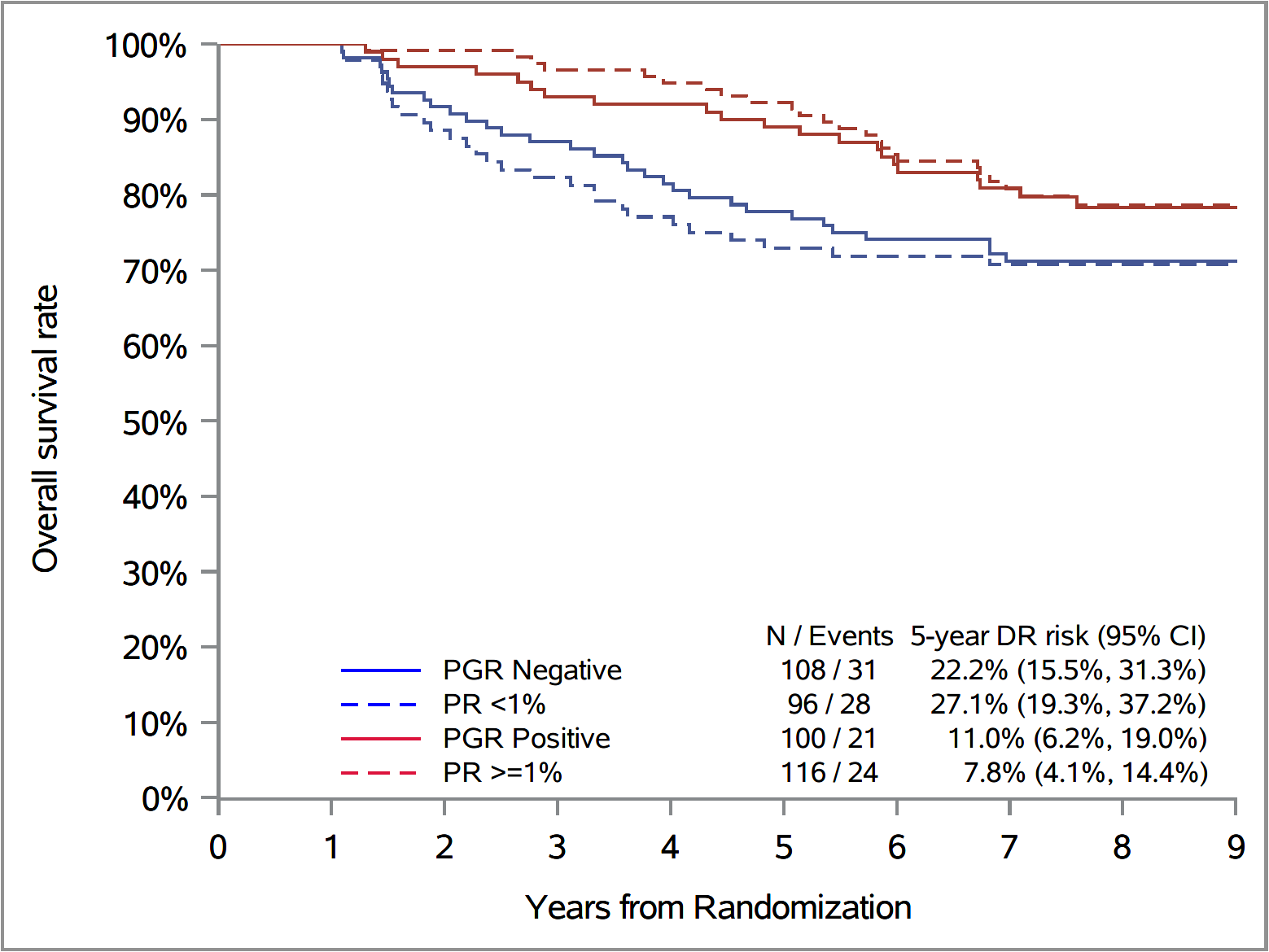

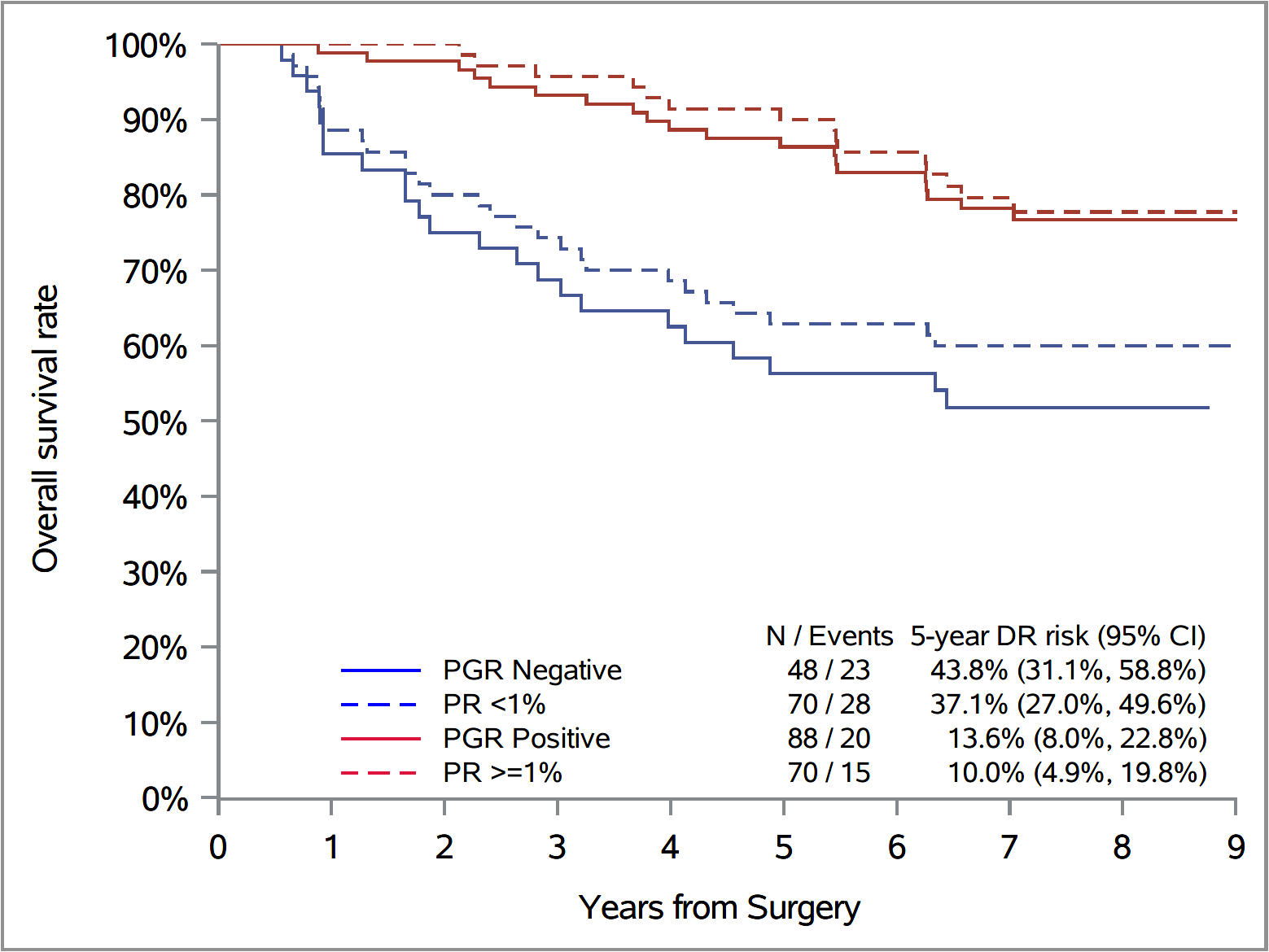


E F


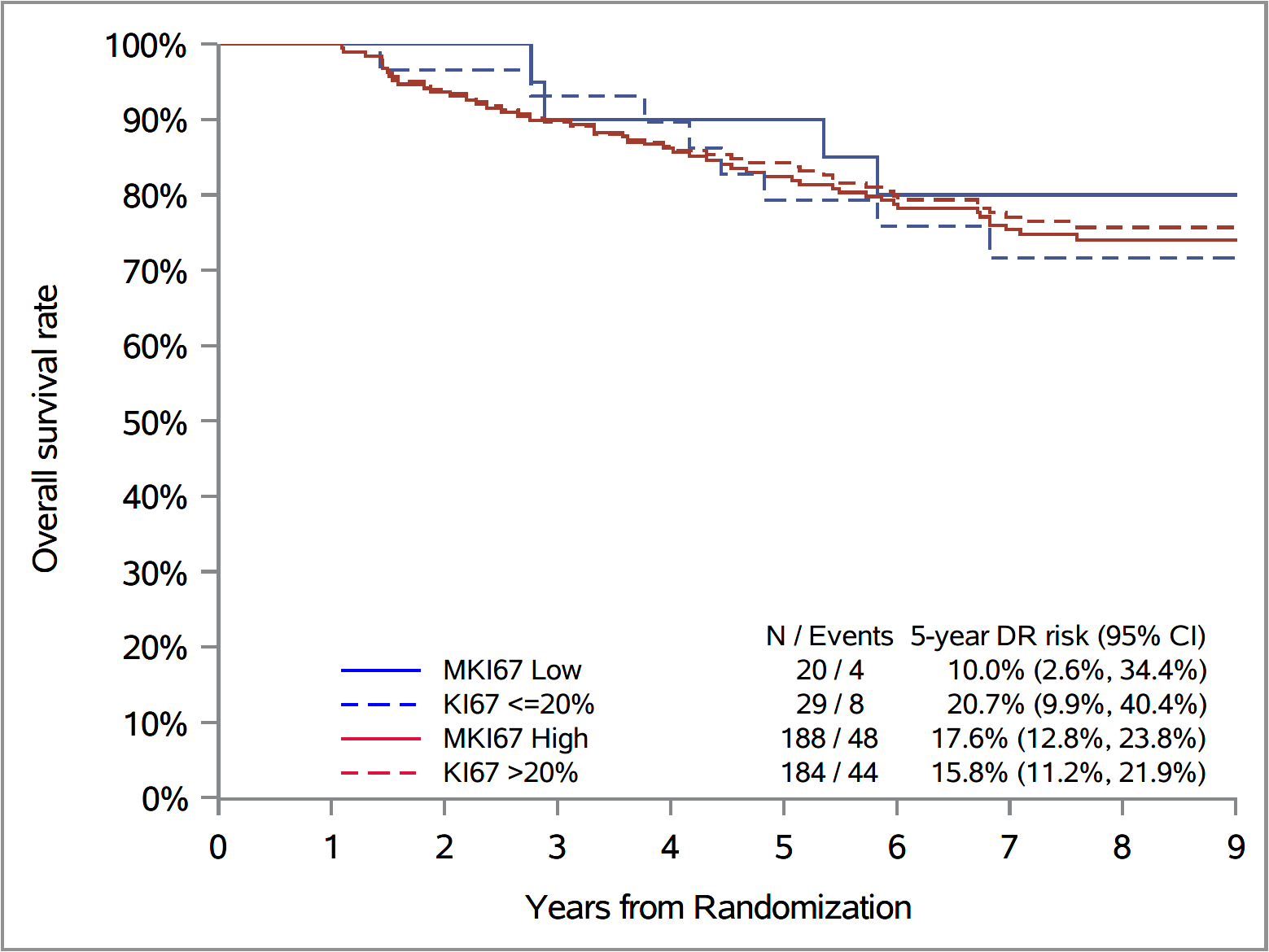

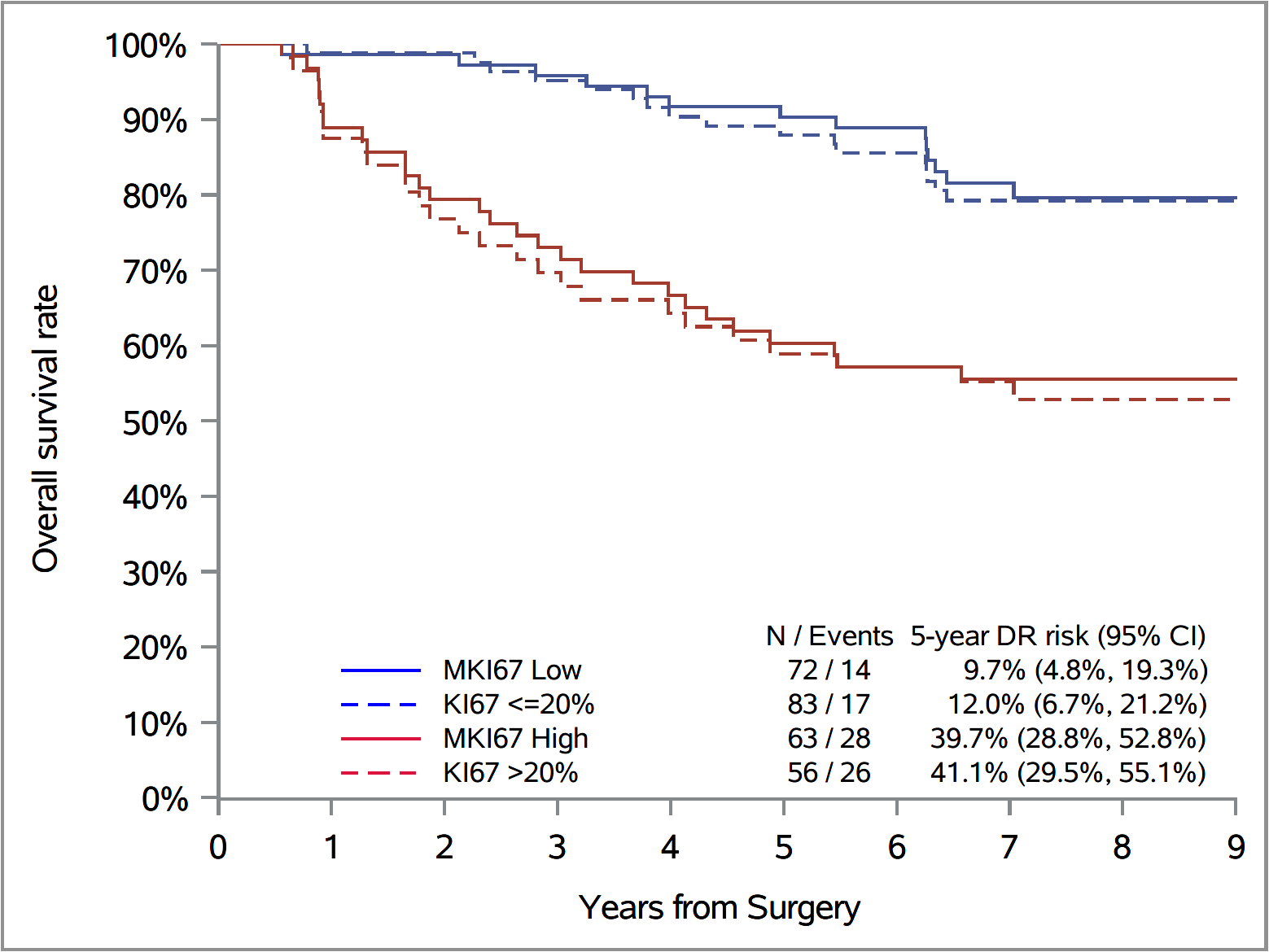

Supplement: Multimedia component 1 [file mmc1.docx]
